# Atherogenic Index of Plasma is Independently Associated With Microalbuminuria in Type 2 Diabetes: A Retrospective Cross‐Sectional Study

**DOI:** 10.1155/jdr/5461854

**Published:** 2026-09-08

**Authors:** Yaqin Liu, Xiaoyu Liu, Shujie Zhao, Qingling Bian, Shan Wang, Yanyan He, Fangxin Mu, Yun Feng

**Affiliations:** ^1^ Tuberculosis Hospital of Shaanxi Province(The Fifth People′s Hospital Of Shaanxi Province),, Xi′an, China; ^2^ The Second Affiliated Hospital of Jilin University, Changchun City, Jilin Province, China, jlu.edu.cn; ^3^ The First Affiliated Hospital of Xi′an Jiaotong University, Xi′an, Shaanxi, China, xjtu.edu.cn

**Keywords:** atherogenic index of plasma, cross-sectional study, diabetic kidney disease, microalbuminuria, risk factor, Type 2 diabetes

## Abstract

**Background:**

The atherogenic index of plasma (AIP) reflects the balance between proatherogenic and antiatherogenic lipoproteins and is a novel marker of atherosclerosis.

**Objectives:**

This study is aimed at investigating the association between AIP and microalbuminuria in T2DM.

**Methods:**

This retrospective cross‐sectional study included 254 patients with Type 2 diabetes mellitus divided into normoalbuminuria group and microalbuminuria group. Models were constructed for regression analysis. Restricted cubic spline was used to analyze the dose‐response relationship between AIP and UACR. ROC curve was used to evaluate the discrimination power. The integrated discrimination improvement (IDI) was used to evaluate the incremental value of adding AIP to the base model.

**Results:**

AIP levels were significantly higher in the microalbuminuria group than in the normal albuminuria group (0.32 ± 0.41 vs. 0.19 ± 0.33, *p* = 0.005). After adjusting for sex, age, blood pressure, BMI, HbA1c, and eGFR, elevated AIP remained independently associated with microalbuminuria (OR = 3.291, 95% CI: 1.411–7.676, *p* = 0.006). A significant dose‐response trend was observed across AIP quartiles (P for trend = 0.010), with the highest quartile showing a 3.34‐fold increased risk compared with the lowest (OR = 3.344, 95% CI: 1.397–8.006, *p* = 0.007). HbA1c (OR = 1.330) and eGFR (OR = 0.970) were also independent risk factors. The discriminative performance of AIP alone was modest, and its incremental value over conventional risk factors was limited.

**Conclusion:**

AIP is independently associated with microalbuminuria in T2DM with a significant dose‐response relationship, suggesting its potential as an adjunctive risk marker for early DKD in primary care. However, its modest discriminative ability precludes use as a standalone screening tool. These hypothesis‐generating findings require prospective validation.

## 1. Introduction

Diabetic kidney disease (DKD) is a devastating microvascular complication of Type 2 diabetes mellitus (T2DM) and a leading cause of end‐stage renal disease worldwide [1]. Microalbuminuria is one of the criteria for the diagnosis of diabetic nephropathy. Early detection at the microalbuminuria stage is critical for implementing interventions that can slow disease progression [2]. However, the diagnosis of DKD requires more measurement of urinary microalbuminuria, and, conventional markers such as urinary albumin excretion have limitations in sensitivity and specificity for incipient DKD, because of its limitations, the complexity of testing, and the need to intervene earlier, before microalbuminuria develops, there is a need to identify more clinically available risk markers associated with microalbuminuria [3].

Beyond chronic hyperglycemia, dyslipidemia plays a pivotal role in DKD pathogenesis. “Lipotoxicity,” driven by atherogenic lipid profiles, contributes to renal injury via inflammation, oxidative stress, and endothelial dysfunction [4–6]. The atherogenic index of plasma (AIP), calculated as log_10_(triglycerides[TG]/HDL‐cholesterol), is a composite lipid metric that reflects the balance between proatherogenic and antiatherogenic lipoproteins [7]. A high AIP correlates strongly with the presence of small, dense low‐density lipoprotein (sdLDL) particles, which are highly atherogenic, and with an impaired reverse cholesterol transport [8] [9]. AIP is a validated marker of cardiovascular risk [10].

Previous cross‐sectional and cohort studies have established a significant association between the AIP and the prevalence of albuminuria or diabetic nephropathy in general diabetic populations. For instance, Yan et al. classified AIP by tertiles and found that the highest tertile group of AIP was significantly related to the occurrence of diabetic nephropathy, and the same results were found after studying AIP as a continuous variable (all *p* < 0.05) [11]. The study by Zhang et al. found a continuous positive association between AIP and the risk of diabetic nephropathy, and this association remained statistically significant after comprehensive adjustment for multiple confounding factors [12]. A meta‐analysis by Min et al. supported a significant association between elevated AIP and DN in patients with T2DM, finding that individuals with higher AIP levels had a 51% higher risk of DNP than those with lower levels, and this association was consistently observed across multiple subgroups [13]. Sahani et al. similarly found an association between AIP and diabetic nephropathy [14]. The research conducted by Wei et al. revealed that the increase of AIP index is an associated factor for patients with early‐onset Type 2 diabetic nephropathy [15]. The above studies have demonstrated a correlation between AIP and diabetic nephropathy. Qi et al. reported that AIP predicted microalbuminuria in newly diagnosed T2DM patients [16]. However, this study did not assess the reclassification ability and comprehensive discrimination improvement of AIP.

However, these findings were predominantly derived from cohorts with a high proportion of lipid‐lowering or antihypertensive medication use, which may have substantially attenuated the native relationship between AIP and renal damage. Furthermore, although the association is recognized, the incremental predictive utility of incorporating AIP into conventional risk models—specifically, whether it significantly improves the reclassification of individuals beyond traditional factors—remains largely unexplored. Therefore, this study is aimed at addressing these gaps by (1) evaluating this relationship in a relatively treatment‐naive population to minimize confounding by drug interventions, and (2) employing integrated discrimination improvement (IDI) and net reclassification improvement (NRI) analyses to quantify the clinical net benefit of adding AIP to the established risk prediction model. (3) By mandatory inclusion, clinically significant traditional risk factors were included in the model for regression analysis, and stratified analysis was performed according to the quartile of AIP to evaluate the dose‐response relationship between AIP and urinary albumin‐to‐creatinine ratio (UACR), and restricted cubic spline (RCS) curve was drawn to evaluate the nonlinear relationship. (4) Subgroup analysis was performed according to gender and obesity status to determine whether the effect of AIP was consistent in different populations.

Given the shared pathways of endothelial injury in atherosclerosis and DKD. This cross‐sectional study is aimed at investigating the association between AIP and microalbuminuria, and at evaluating whether AIP provides incremental risk information beyond traditional risk factors. Although UACR is the gold standard for detecting early DKD, its measurement requires specialized equipment and may not be universally available in primary care or resource‐limited settings. Identifying a simple, calculable marker derived from routine lipid profiles could serve as an initial triage tool to prioritize high‐risk individuals for confirmatory UACR testing, thereby facilitating earlier detection and intervention.

## 2. Materials and Methods

### 2.1. Study Design and Participants

This retrospective cross‐sectional study was conducted at the Department of Endocrinology, the Second Hospital of Jilin University. A consecutive sample of 254 patients diagnosed with T2DM who were admitted between April 2021 and December 2022 was included. This study has been approved by the Ethics Committee of the Second Hospital of Jilin University (Approval No. 2024 Research Review 121). Due to the retrospective nature of the research, the requirement for informed consent was waived.

### 2.2. Inclusion and Exclusion Criteria

Patients were eligible if they were aged between 18 and 75 years, had a confirmed diagnosis of T2DM according to the Chinese Guidelines for the Prevention and Treatment of Type 2 Diabetes (2020 edition), and had complete clinical and laboratory records available for analysis. Additionally, only patients with no history of lipid‐lowering medication use prior to blood sampling were included to ensure that lipid profiles reflected untreated metabolic status

Participants were excluded if they had Type 1 diabetes, gestational diabetes, latent autoimmune diabetes, or other specific forms of diabetes. We also excluded individuals with acute diabetic complications (e.g., ketoacidosis or hyperosmolar hyperglycemic state), primary or secondary renal diseases unrelated to diabetes, significant cardiac or hepatic dysfunction, malignancy, active infectious diseases, or those who were pregnant or lactating. To minimize confounding effects on renal and metabolic parameters, patients who had taken angiotensin‐converting enzyme inhibitors (ACEIs), angiotensin II receptor blockers (ARBs), sodium‐glucose cotransporter‐2 inhibitors (SGLT2i), glucagon‐like peptide‐1 receptor agonists (GLP‐1RAs), or lipid‐lowering agents within the preceding 3 months were excluded.

### 2.3. Group Definition

Based on the Chinese Guidelines for DKD (2021 edition), early DKD was defined as a UACR between 30 and 300 mg/g. Participants were categorized into two groups: the normal albuminuria group (NAG) (UACR < 30 mg/g)(155) and the microalbuminuria group (MAG) (UACR 30–300 mg/g)(99).

According to the guidelines for the prevention and treatment of DKD in China (2021 edition), the diagnostic criteria for diabetes is as follows: within 3–6 months after excluding interfering factors UACR ≥ 30 mg/g or UAER ≥ 30 mg/24 h (≥ 20 *μ*g/min), or estimated glomerular filtration rate (eGFR) < 60 mL·min^−1^·(1.73 m^2^)^−1^ were performed for more than 3 months at least two of the three tests, or renal biopsy consistent with DKD pathological changes. The diagnosis of Type 2 DKD is based on the presence of either element. In this study, the results of UACR were collected only once and only the relationship between AIP and UACR was investigated.

### 2.4. Data Collection

Demographic information (age, sex), anthropometric measures (height, weight, systolic and diastolic blood pressure [SBP and DBP]), and laboratory data were extracted from electronic medical records. Body mass index (BMI) was calculated as weight in kilograms divided by height in meters squared (kg/m^2^).

Fasting venous blood samples were analyzed for glucose, lipid profiles, renal function markers, and other biochemical parameters. Measured variables included fasting plasma glucose (FPG), glycated hemoglobin (HbA1c), fasting insulin (FINS), TG, total cholesterol (TC), high‐density lipoprotein cholesterol (HDL‐C), low‐density lipoprotein cholesterol (LDL‐C), serum creatinine (Scr), blood urea nitrogen (BUN), uric acid (UA), cystatin C (Cys‐C or Cyc), *α*1‐microglobulin (*α*1‐MG), *β*2‐microglobulin (*β*2‐MG), retinol‐binding protein (RBP), and homocysteine (Hcy). eGFR was calculated using the Chronic Kidney Disease Epidemiology Collaboration (CKD‐EPI) equation. Urinary markers included urinary microalbumin (MA), urinary creatinine (Ucr), and the albumin‐to‐creatinine ratio (UACR). Although clinical guidelines recommend repeated measurements for diagnosis, our study utilized a single UACR measurement due to the nature of the study design. The AIP was computed as the base‐10 logarithm of the ratio of TG to HDL‐C (both in mmol/L): log_10_(TG/HDL‐cholesterol).

### 2.5. Statistical Analysis

Continuous variables were assessed for normality using the Shapiro–Wilk test. Normally distributed data were expressed as mean ± standard deviation and compared between groups using the independent‐samples *t*‐test. Nonnormally distributed variables were summarized as median (interquartile range) and compared with the Mann–Whitney *U* test. Categorical variables were presented as frequencies (percentages) and analyzed using the chi‐square test.

Correlations between UACR (or AIP) and other clinical parameters were examined using Spearman′s rank‐order correlation. To identify independent risk factors for microalbuminuria, variables with *p* < 0.10 in univariate analyses or deemed clinically relevant were entered into a multivariate binary logistic regression model using forward stepwise selection (likelihood‐ratio criterion). Considering that stepwise regression identifies statistically significant dependent variables does not necessarily control for biologically important confounders. Therefore, the traditional risk factors with clinical significance were included in the model for regression analysis by mandatory inclusion, and the stratified analysis was performed according to the quartile of AIP to evaluate the dose‐response relationship between AIP and UACR. The nonlinear relationship between AIP and UACR was fitted by RCS. Subgroup analysis was performed according to gender and obesity status to determine whether the effect of AIP was consistent in different populations. Multicollinearity was evaluated with variance inflation factors (VIF < 5 considered acceptable). Results were reported as odds ratios (ORs) with 95% confidence intervals (CIs).

To evaluate whether AIP provides incremental predictive value beyond conventional lipid parameters, we performed nested model comparisons using three separate multivariate logistic regression models, each containing one lipid parameter (TG, HDL‐C, or AIP) along with the same set of seven covariates. Model performance was compared using the Akaike information criterion (AIC) and Nagelkerke R [2], with lower AIC values indicating better model fit [17].

The discriminative ability of AIP alone and of the combined model was assessed using the area under the receiver operating characteristic curve (AUC‐ROC). For AIP as a standalone predictor, the optimal cutoff value was determined by maximizing the Youden index, and sensitivity, specificity, positive predictive value (PPV), and negative predictive value (NPV) were reported.

The incremental predictive value of AIP was evaluated by comparing the AUC of a base model (containing FPG and Cys‐C) with that of an enhanced model (base model + AIP) using DeLong′s test. Additionally, NRI and IDI were calculated. A two‐tailed *p* value < 0.05 was considered statistically significant.

All analyses were performed using SPSS Version 26.0 (IBM Corp., Armonk, New York, United States) and R Version 4.3.1 (R Foundation for Statistical Computing, Vienna, Austria).

## 3. Results

### 3.1. Baseline Characteristics

Among the 254 enrolled T2DM patients, 155 (61.0%) were classified into the NAG and 99 (39.0%) into the MAG. The baseline characteristics are summarized in Table 1.

**Table 1:** Baseline characteristics of T2DM patients with normal albuminuria and microalbuminuria.

Project	Normal group (*n* = 155)	Microalbuminuria group (*n* = 99)	*p*
Gender (%)			
Male	88 (56.8%)	57 (57.6%)	0.9
Female	67 (43.2%)	42 (42.4%)	
Age (years)	48.92 ± 13.04	47.32 ± 13.51	0.35
SBP (mmHg)	126.90 ± 9.82	126.48 ± 11.29	0.759
DBP (mmHg)	80.54 ± 6.46	80.52 ± 8.65	0.983
Height (m)	1.68 ± 0.08	1.68 ± 0.08	0.918
Weight (kg)	72.05 ± 12.92	71.75 ± 15.46	0.866
BMI (kg/m2)	25.58 ± 3.74	25.34 ± 4.23	0.43
FBG (mmol/L)	8.26 (6.68, 11.16)	10.77 (8.15, 14.21)	< 0.001 ^∗∗^
HbA1c (%)	8.61 ± 1.89	9.65 ± 2.28	< 0.001 ^∗∗^
FINS (uIU/mL)	13.30 (8.65, 19.82)	12.58 (7.29, 20.56)	0.656
TG (mmol/L)	1.63(1.09, 2.69)	2.05 (1.31, 3.17)	0.015 ^∗^
TC (mmol/L)	5.43 ± 1.23	6.10 ± 1.58	< 0.001 ^∗∗^
LDL‐C (mmol/L)	3.02 ± 0.92	3.15 ± 0.98	0.293
HDL‐C (mmol/L)	1.17 ± 0.29	1.10 ± 0.28	0.085
AIP	0.19 ± 0.33	0.32 ± 0.41	0.005 ^∗∗^
Scr (umol/L)	63 (51, 74)	66 (55, 81)	0.055
BUN (mmol/L)	5.07 (4.39, 6.14)	5.41 (4.73, 6.63)	0.025 ^∗^
UA (umol/L)	315.81 ± 83.63	342.99 ± 96.25	0.018
eGFR (mL/min)	105.25 ± 17.95	99.86 ± 27.14	0.058
*α*1‐MG (mg/L)	22.03 (19.0, 26.0)	25.10 (21.0, 30.0)	< 0.001 ^∗∗^
*β*2‐MG (mg/L)	1.66 (1.48, 1.83)	1.78 (1.53, 2.24)	0.003 ^∗∗^
RBP (mg/L)	35.0 (27.0, 51.0)	35.0 (30.0, 45.0)	0.73
Cys‐C (mg/L)	0.67 (0.58, 0.77)	0.68 (0.57, 0.88)	0.088
Hcy (umol/L)	10.0 (8.0, 13.0)	10.0 (9.0, 14.0)	0.753
MA (mg/L)	10.0 (10.0, 10.0)	80.0 (80.0, 150.0)	< 0.001 ^∗∗^
UCr (mg/dL)	300.0 (200.0, 300.0)	100.0 (50.0, 200.0)	< 0.001 ^∗∗^
UACR (mg/g)	0.05 (0.03, 0.20)	0.75 (0.50, 1.60)	< 0.001 ^∗∗^

*Note:* The asterisk “∗” means *p* < 0.05; “∗∗” means *p* < 0.01.

There were no statistically significant differences in gender, age, blood pressure, height, weight, or BMI between the two groups. Regarding metabolic and renal parameters, the MAG exhibited significantly higher levels of FPG, HbA1c, TG, AIP, BUN, UA, *α*1‐MG, *β*2‐MG, and UMA (all *p* < 0.05). UCr levels were significantly lower in the MAG (*p* < 0.05). Notably, AIP levels were significantly elevated in the MAG compared with the NAG (0.32 ± 0.41 vs. 0.19 ± 0.33; *p* < 0.01).

### 3.2. Correlation Analyses

Spearman correlation analysis revealed that AIP was positively correlated with diastolic pressure, BMI, FPG, FINS, TG, TC, Scr, UA, eGFR, *α*1‐MG, RBP, Hcy, UMA, and UACR (all *p* < 0.05), and negatively correlated with age, LDL‐C and HDL‐C (*p* < 0.01). UACR showed significant positive correlations with FBG, HbA1c, TG, TC, AIP, BUN, *α*1‐MG, *β*2‐MG, and Cys‐C (all *p* < 0.05) (Table 2).

**Table 2:** Correlation analysis of each variable with UACR and AIP.

Variate	*r*value of UACR	*p*value of UACR	*r* value of AIP	*p* value of AIP
Gender (%)	−0.04	0.522	0.282	< 0.001 ^∗∗^
Age (years)	−0.021	0.735	‐0.294	< 0.001 ^∗∗^
SBP (mmHg)	−0.034	0.593	0.117	0.063
DBP (mmHg)	−0.085	0.176	0.148	0.019 ^∗^
BMI (kg/m2)	−0.019	0.768	0.27	< 0.001 ^∗∗^
FBG (mmol/L)	0.314	< 0.001 ^∗∗^	0.175	0.005 ^∗∗^
HbA1c (%)	0.287	< 0.001 ^∗∗^	0.071	0.263
TG (mmol/L)	0.161	0.01 ^∗^	0.955	< 0.001 ^∗∗^
TC (mmol/L)	0.223	< 0.001 ^∗∗^	0.313	< 0.001 ^∗∗^
LDL‐C (mmol/L)	0.091	0.15	‐0.215	0.001 ^∗∗^
HDL‐C (mmol/L)	−0.078	0.214	‐0.657	< 0.001 ^∗∗^
AIP	0.167	0.008 ^∗∗^	——	——
Scr (umol/L)	0.048	0.447	0.136	0.03 ^∗^
eGFR (mL/min)	−0.067	0.288	0.127	0.044 ^∗^
Cys‐C(mg/L)	0.132	0.036 ^∗^	0.023	0.714
UACR(mg/g)	——	——	0.167	0.008 ^∗∗^

*Note:* This table presents only a few indicators that are most strongly correlated or clinically relevant with the core variables (AIP, UACR); see Supporting Information 1 for the full correlation matrix table. The asterisk ”∗” means *p* < 0.05; “∗∗” means *p* < 0.01.

### 3.3. Logistic Regression Analysis for Microalbuminuria Risk

In univariate logistic regression, HbA1c, FPG, AIP, BUN, Cys‐C, and Ucr were significant risk factors of microalbuminuria (all *p* < 0.05). These variables were entered into the multivariate model. Given the direct mathematical relationship between Ucr and UACR, a secondary model excluding Ucr was constructed. The final multivariate analysis identified three independent risk factors for microalbuminuria: elevated FPG (OR = 1.214, 95% CI: 1.120–1.316, *p* < 0.001), elevated Cys‐C (OR = 7.995, 95% CI: 2.258–28.312, *p* = 0.001), and elevated AIP (OR = 2.315, 95% CI: 1.063–5.043, *p* = 0.03) (Table 3).

**Table 3:** Multivariate logistic regression analysis for microalbuminuria (after excluding Ucr).

Variate	OR	95% CI	*p*
FBG (mmol/L)	1.214	1.120–1.316	< 0.001
AIP	2.315	1.063–5.043	0.03
Cys‐C (mg/L)	7.995	2.258–28.312	0.001

To evaluate the independent association between AIP and microalbuminuria while controlling for potential confounders, we constructed a multivariable logistic regression model using the enter method, simultaneously including the following variables: sex (male/female, with female as reference), age, SBP (mmHg), DBP (mmHg), BMI (kg/m^2^), HbA1c (%), eGFR (mL/min/1.73m^2^), and AIP. All variables were forced into the model without stepwise selection. All VIF values were < 5, ranging from 1.065 to 1.880, indicating no significant collinearity.

In the multivariate logistic regression analysis included all traditional risk factors, AIP remained independently associated with microalbuminuria (OR = 3.291, 95% CI: 1.411–7.676, *p* = 0.006). The risk of microalbuminuria increased approximately 2.3‐fold for each 1‐unit increase in AIP. HbA1c (OR = 1.334, 95% CI: 1.161–1.533, *p* < 0.001) and eGFR (OR = 0.970, 95% CI: 0.953–0.987, *p* < 0.001) were also significantly correlated with microalbuminuria, whereas gender, age, blood pressure, and BMI did not reach statistical significance (all *p* > 0.05) (Table 4).

**Table 4:** Multivariable logistic regression analysis with forced entry of all traditional risk factors for microalbuminuria.

Variable	OR	95% CI	*p*
Sex (male vs. female)	0.75	0.42–1.35	0.341
Age (years)	0.97	0.94–1.00	0.068
SBP (mmHg)	0.99	0.96–1.03	0.627
DBP (mmHg)	1.00	0.96–1.05	0.892
BMI (kg/m^2^)	0.96	0.89–1.04	0.3
HbA1c (%)	1.33	1.16–1.53	< 0.001
eGFR (mL/min)	0.97	0.95–0.99	< 0.001
AIP	3.29	1.41–7.68	0.006

### 3.4. One‐Way ANOVA Between Quartiles of AIP

Among different quartiles of AIP (Q1–Q4), one‐way ANOVA results showed that, age (*p* < 0.001), height (*p* < 0.001), weight (*p* < 0.001), BMI (*p* < 0.001), TC (*p* = 0.003), LDL‐C (*p* < 0.001), UA (*p* < 0.001), RBP (*p* = 0.002), Hcy (*p* = 0.010), MA (*p* = 0.039), and UACR (*p* = 0.045) between the two groups were statistically significant. All the above indexes showed an increasing trend with the increase of AIP. However, there were no significant differences in SBP, DBP, fasting blood glucose (*p* = 0.053), glycosylated hemoglobin, fasting islet hormone, Scr, BUN, eGFR, *α*1‐MG, *β*2‐MG, Cys‐C, and urine creatinine among the three groups (all *p* > 0.05). The above details are provided in the Supporting Information 2.

### 3.5. Multivariate Logistic Regression Analysis of Microalbuminuria Related Factors After AIP Grouped

Multivariable logistic regression analysis (Table 5) showed that, after adjusting for sex, age, SBP, DBP, BMI, HbA1C, and eGFR, the overall association between AIP quartiles and the outcome was of borderline statistical significance (Wald *χ* ^2^ = 7.398, *p* = 0.060). Compared with Q1, Q4 was associated with a significantly increased risk of the outcome (OR = 3.344, 95% CI: 1.397–8.006, *p* = 0.007), whereas Q2 (OR = 1.888, 95% CI: 0.829–4.300, *p* = 0.130) and Q3 (OR = 1.901, 95% CI: 0.812–4.447, *p* = 0.139) did not reach statistical significance.

**Table 5:** Multivariate logistic regression analysis of microalbuminuria related factors after AIP grouped.

Variable	OR	(95% CI)	*p*
AIP quartiles			
Q1		1.00 (Ref)	Ref
Q2	1.888	0.829–4.300	0.13
Q3	1.901	0.812–4.447	0.139
Q4	3.344	1.397–8.006	0.007
P for trend	1.431	1.088–1.883	0.01
Covariates			
Sex (male vs. female)	1.333	0.743–2.392	0.336
Age	0.973	0.945–1.003	0.073
SBP	0.992	0.959–1.026	0.646
DBP	1.003	0.958–1.051	0.884
BMI	0.964	0.893–1.040	0.342
HbA1C	1.330	1.158–1.526	< 0.001
eGFR	0.970	0.953–0.987	0.001

*Note:* Adjusted for sex, age, SBP, DBP, BMI, HbA1C, and eGFR. AIP was categorized into quartiles with Q1 as the reference. P for trend was calculated by treating the quartile categories as a continuous ordinal variable (assigned values 1, 2, 3, and 4).

When the AIP quartile categories were entered as an ordinal variable in the regression model, a significant dose‐response relationship was observed between AIP levels and the outcome (P for trend = 0.010), with each one‐level increase in AIP quartile associated with a 43.1% higher risk of the outcome (OR = 1.431, 95% CI: 1.088–1.883).

Additionally, HbA1C (OR = 1.330, 95% CI: 1.158–1.526, *p* < 0.001) and eGFR (OR = 0.970, 95% CI: 0.953–0.987, *p* = 0.001) were identified as independent risk factors of the outcome.

### 3.6. Subgroup Analyses

In sex‐stratified analyses, the association between AIP and UACR was statistically significant in females (overall *p* = 0.037), with Q2 (OR = 4.423, 95% CI: 1.317–14.855, *p* = 0.016) and Q3 (OR = 5.330, 95% CI: 1.449–19.603, *p* = 0.012) showing significantly increased risks, whereas Q4 showed a borderline significant association (OR = 3.476, 95% CI: 0.915–13.201, *p* = 0.067). In males, no significant association was observed across AIP quartiles (overall *p* = 0.135). However, the test for interaction between AIP and sex did not reach statistical significance (P for interaction = 0.506), suggesting that the effect of AIP on the outcome was not significantly modified by sex. Details of the above are provided in the Supporting Information 3.

In obesity‐stratified analyses, among obese participants (BMI ≥ 24), the highest AIP quartile was associated with a significantly increased risk of microalbuminuria compared with the lowest quartile (OR = 4.546, 95% CI: 1.272–16.249, *p* = 0.020), whereas in nonobese participants, the association was of borderline significance (OR = 3.730, 95% CI: 0.900–15.462, *p* = 0.070). However, the test for interaction between AIP and obesity status was not significant (P for interaction = 0.820), indicating that the effect of AIP on UACR was not significantly modified by BMI. Details of the above are provided in the Supporting Information 4.

### 3.7. RCS Analysis

To further explore the shape of the association between AIP and UACR, we performed RCS analysis with 4 knots, adjusting for the same covariates as in the main model. The overall association was statistically significant (*χ* ^2^ = 8.63, df = 3, *p* = 0.0346). The test for nonlinearity was not significant *χ* ^2^ = 1.66, df = 2, *p* = 0.4362), indicating that the relationship was adequately captured by a linear dose‐response trend (Supporting Information 5). This result is mutually consistent with the P for trend = 0.010 found in the previous quartile analysis, and together supports a significant linear dose‐response relationship between AIP and UACR.

### 3.8. Nested Model Comparison

As shown in Supporting Information 6, the model containing TG demonstrated the lowest AIC (315.148) and the highest Nagelkerke *R* ^2^ (0.200), followed by the AIP model (AIC = 317.727, *R* ^2^ = 0.188) and the HDL‐C model (AIC = 323.612, *R* ^2^ = 0.161). The *Δ*AIC between TG and AIP was 2.58, indicating comparable predictive performance between the two indices. Importantly, AIP offers the practical advantage of being a single composite indicator that integrates both proatherogenic (TG) and antiatherogenic (HDL‐C) lipid profiles, eliminating the need to interpret multiple separate parameters. This feature may be particularly valuable in clinical settings requiring rapid, integrated risk stratification.

### 3.9. Discriminative Ability of AIP and the Combined Model

The ROC curve for AIP alone in predicting microalbuminuria yielded an AUC of 0.595 (95% CI: 0.523–0.667, *p* = 0.015) (Supporting Information 7). The optimal cutoff value was 0.0397, with a sensitivity of 79.8%, specificity of 36.7%, PPV of 44.6%, and NPV of 36.3%.

A combined model incorporating the seven independent predictors (gender + age + DBP + SBP + HbA1c + BMI + eGFR + AIP) demonstrated an AUC of 0.712 (95% CI:0.647–0.776, *p* > 0.001) (Supporting Information 9). The predicted probability of the model was highly consistent with the observed probability, and the calibration was good (mean absolute error = 0.031, mean squared error = 0.00129, 0.9 quantile of absolute error = 0.057) (Supporting Information 11). For exploratory purposes, a nomogram was also developed (Supporting Information 10).

**Figure 1:**
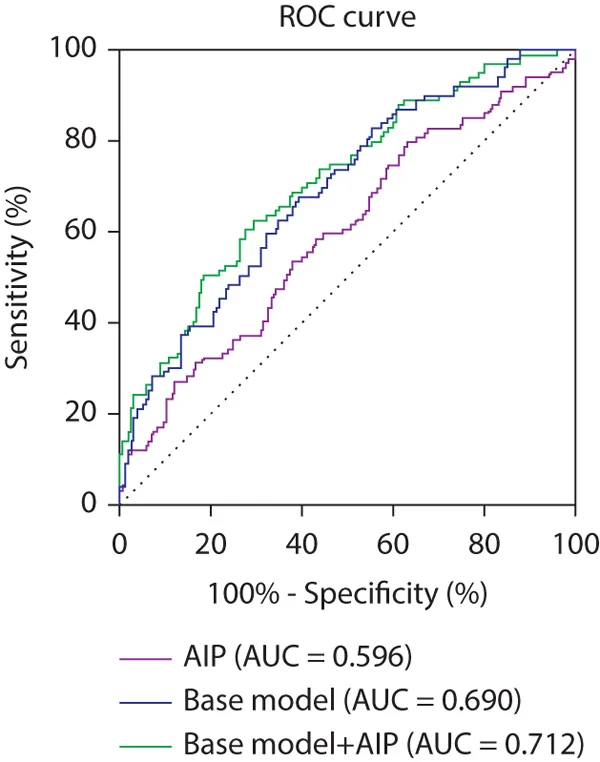
Receiver operating characteristic (ROC) curves of AIP alone, the base model (Gender + Age + DBP + SBP + HbA1c + BMI + eGFR), and the combined model (Gender + Age + DBP + SBP + HbA1c + BMI + eGFR + AIP) for predicting microalbuminuria in T2DM patients. Abbreviations: AIP, atherogenic index of plasma; FPG, fasting plasma glucose; Cys‐C, cystatin C; AUC, area under the curve.

To assess the incremental value of AIP, a base model containing gender, age, DBP, SBP, HbA1c, BMI, and eGFR achieved an AUC of 0.690 (95% CI: 0.624–0.756, *p* < 0.001) (Supporting Information 8). Adding AIP to this base model significantly improved the AUC to 0.712 (95% CI: 0.647–0.776, *p* < 0.001) (Supporting Information 9) (*P* for comparison = 0.0047) (DeLong′s test). The IDI was 0.0291 (*p* = 0.0096) indicating a small but statistically significant improvement in overall risk discrimination. The NRI was 0.0166 (95% CI: −0.108~0.144), which did not reach statistical significance. Three ROC curves have been plotted in a figure (Figure 1). The diagonal dashed line represents the reference line (AUC = 0.5). The combined model achieved the highest AUC (0.712), followed by the base model (0.690) and AIP alone (0.595). The difference between the combined model and the base model was statistically significant (P for comparison = 0.0047).

## 4. Discussion

This cross‐sectional study demonstrates that the AIP is independently associated with microalbuminuria in patients with T2DM. After adjusting for gender, age, DBP, SBP, HbA1c, BMI, and eGFR fasting blood glucose and Cys‐C, AIP remained a significant risk factor (OR = 3.291, 95% CI: 1.411–7.676, *p* = 0.006) (OR = 2.315, 95% CI: 1.063–5.043, *p* = 0.03). This finding has potential relevance for primary care settings where UACR measurement may not be routinely available. AIP, calculated from standard lipid panels, could serve as an accessible and cost‐free initial risk marker to identify individuals who might benefit from targeted UACR screening, rather than replacing the gold standard. However, the modest discriminative ability (AUC = 0.595 for AIP alone) and low specificity (36.7%) indicate that AIP should not be used as a standalone screening test for early DKD. Instead, it may serve as an adjunctive risk marker that, when combined with gender, age, DBP, SBP, HbA1c, BMI, and eGFR FBG and Cys‐C, modestly improves risk discrimination (IDI = 2.91*%* improvement, *p* = 0.0096) (IDI = 1.5*%* improvement, *p* = 0.04). The nonsignificant NRI (0.0166, *p* > 0.05) (0.051, *p* = 0.170) suggests that the reclassification improvement may not be clinically meaningful at the risk thresholds used in this study, which may be due to the limited sample size or the specific cutoff values chosen.

The low specificity of AIP (36.7%) at the optimal cutoff of 0.0397 implies that using AIP alone to screen for microalbuminuria would result in a high false‐positive rate (63.3%), leading to unnecessary anxiety and further testing. However, as a component of a multimarker panel, AIP may add modest value. The significant IDI (2.9% improvement) suggests that AIP provides a small but statistically significant improvement in risk discrimination, which might be useful in research settings or for refining risk estimates in intermediate‐risk populations.

This study found that the risk of AIP in the highest quartile group (Q4) was 3.34 times that of the lowest quartile group (Q1), and there was a significant dose‐response trend (P for trend = 0.010), suggesting that the increase in AIP levels is closely related to the increased risk of outcome events. Although the OR values for Q2 and Q3 groups were both > 1 (1.888 and 1.901, respectively), they did not reach statistical significance, which might be related to the limited sample size or might indicate that the pathogenic effect of AIP is mainly significant at higher levels (above a certain threshold)—that is, there is a “threshold effect.” Previous studies have shown that AIP is positively correlated with the risk of microalbuminuria in diabetic patients. The results of this study are consistent with this and further clarify the risk stratification value of AIP in this population. AIP, as a simple and cost‐effective lipid‐derived indicator, can be used to identify high‐risk individuals. In this study, the risk of disease in the Q4 group (with the highest AIP level) was significantly increased, suggesting that AIP can be included in the routine risk assessment system in clinical practice, and key screening and early intervention should be carried out for the Q4 population.

Although the sex‐stratified analysis suggested a more pronounced association between AIP and UACR in females than in males, the formal test for interaction did not reach statistical significance (P for interaction = 0.506), indicating no significant effect modification by sex.

In our nested model comparisons, the predictive performance of AIP (AIC = 317.727, *R* ^2^ = 0.188) was comparable with that of TG (AIC = 315.148, *R* ^2^ = 0.200), with a modest *Δ*AIC of 2.58, and substantially outperformed HDL‐C alone. This suggests that AIP, as a composite indicator integrating both atherogenic (TG) and antiatherogenic (HDL‐C) lipid profiles, provides a practical and efficient alternative to interpreting multiple separate lipid parameters. This feature may be particularly valuable in clinical settings where rapid, integrated risk stratification is needed. Future mechanistic studies with larger sample sizes may further elucidate the relative contributions of individual lipid components to the observed association, which may inform targeted therapeutic strategies.

Our finding that AIP is independently associated with microalbuminuria aligns with previous reports. Qi et al. found that AIP predicted microalbuminuria in newly diagnosed T2DM patients with an AUC of 0.772 [16]. The slightly lower performance in our study may be attributed to differences in patient characteristics (e.g., longer diabetes duration, broader inclusion of nonnewly diagnosed patients) and our stricter exclusion of patients on lipid‐lowering and renoprotective medications, which may have resulted in a more heterogeneous but also less confounded cohort. Our study extends previous work by quantifying the incremental value of AIP using IDI and by explicitly reporting its low specificity as a limitation.

The link between AIP and renal injury is biologically plausible. A high AIP reflects a predominance of small, dense LDL particles, which are more prone to oxidation and subendothelial retention, promoting foam cell formation and inflammation within the renal microcirculation [18, 19]. Concurrently, low HDL‐C levels imply impaired reverse cholesterol transport and diminished HDL‐mediated vasoprotective functions [4, 5]. This proinflammatory, pro‐oxidant milieu can accelerate glomerulosclerosis and tubulointerstitial fibrosis [20]. Our results support the “lipotoxicity” paradigm in DKD pathogenesis, complementing the classical focus on hyperglycemia. Chen et al. discovered the occurrence of diabetic microvascular lesions is related to dyslipidemia [21], which also indirectly proves the role of lipotoxicity in the development of diabetic nephropathy [22].

At the high end of AIP, it will exacerbate the occurrence of DKD. This is because the increase in TG levels and the accumulation of cholesterol‐rich VLDL remnants, as well as the intensification of residual cholesterol accumulation. These particles are prone to deposit in the vessel walls and activate the formation of macrophage foam cells [23]. Patients with diabetes have an elevated AIP index, indicating a higher ratio of TG to HDL‐C [24], and increased TG can lead to dysfunction of pancreatic beta‐cells and subsequent elevation of blood glucose levels [25]. Reduced HDL‐C also contributes to heightened non‐HDL cholesterol in the liver and bloodstream, resulting in elevated triglyceride levels among patients with diabetes [26]. This vicious cycle will cause the AIP to rise, further promoting the occurrence of atherosclerosis [27]. The research has found that albuminuria is positively correlated with the intensification of systemic and intracellular inflammation, and multiple inflammatory markers are also associated with the presence of early atherosclerosis [28]. Li et al.′s found the interplay between chronic inflammation and endothelial dysfunction in diabetic nephropathy is critical in the pathogenesis of atherosclerosis [29].Inflammatory factors promote the accumulation of foam cells and the formation of atherosclerotic plaques, which aggravates renal and cardiovascular complications [30]. We can infer that the relationship between atherosclerosis and early proteinuria is related to inflammatory factors. Further research can be conducted in the future to verify this relationship.

Apolipoprotein C3 (APOC3) is known to be a key regulator of plasma TG, and current studies suggest a role for APOC3 in the accelerated progression of atherosclerosis and renal disease in Type 1 diabetes. The study by Cervantes et al. found that elevated APOC3 levels at baseline predicted greater loss of renal function [31]. It is suggested that elevated apolipoprotein is related to atherosclerosis in diabetic nephropathy.

Our findings corroborate the previously reported positive association between AIP and albuminuria [16]. Nevertheless, the novelty of our study lies not in merely replicating this association, but in two critical aspects. First, by focusing on a treatment‐naïve cohort, we provide a more robust estimate of the “natural” effect of AIP, largely free from the modifying effects of statins or RAAS blockers, which are common in prior studies. Second, and more importantly, our IDI and NRI results demonstrate that AIP offers a significant NRI beyond the conventional model. Although previous studies primarily relied on ORs to indicate statistical significance, our data suggest that AIP is a meaningful risk stratifier, with a substantial capacity to correctly reclassify intermediate‐risk individuals into more appropriate risk categories. This finding holds practical implications for primary care screening.

However, several limitations must be acknowledged. First, the single‐center, retrospective, cross‐sectional design precludes causal inference. We can only report associations, not predictions of future risk. Second, the diagnosis of early DKD relied on a single UACR measurement; repeated assessments over 3–6 months would be more reliable. Third, the sample size (254 patients, 99 events) is relatively small, which may have limited the statistical power to detect significance in the NRI and may have led to overfitting of the regression model. Fourth, lack of external validation; our findings need confirmation in independent, multicenter cohorts. Fifth, the modest AUC and low specificity of AIP alone limit clinical applicability.

## 5. Conclusion

In this retrospective cross‐sectional study of 254 T2DM patients, the AIP was identified as an independent risk factor for microalbuminuria. When combined with gender, age, DBP, SBP, HbA1c, BMI, and eGFR FBG and Cys‐C, AIP provided a modest but statistically significant improvement in risk discrimination. However, due to its low specificity and modest discriminative ability, AIP alone is not suitable as a standalone screening tool for early DKD. However, as a risk‐screening tool, AIP holds significant importance for primary healthcare screening. Future prospective studies with standardized, repeated albuminuria assessments are essential to confirm whether this association represents a robust predictor of persistent DKD. These hypothesis‐generating findings require validation in prospective, multicenter cohorts before clinical application.

## Author Contributions

Yaqin Liu: conceptualization, data curation, formal analysis, investigation, methodology, software, validation, visualization, writing—original draft, writing—review and editing. Xiaoyu Liu: conceptualization, investigation, methodology, supervision. Shujie Zhao: conceptualization, data curation, formal analysis, methodology, supervision. Qingling Bian: data curation, investigation, software, validation. Shan Wang: data curation, investigation, software, validation. Yanyan He: data curation, investigation, software, validation. Fangxin Mu: conceptualization, data curation, formal analysis, software, validation. Yun Feng: conceptualization, formal analysis, methodology, software, validation, visualization, writing—review and editing.

## Funding

No funding was received for this manuscript.

## Disclosure

All authors have read and approved the final version of the manuscript. Yun Feng had full access to all of the data in this study and takes complete responsibility for the integrity of the data and the accuracy of the data analysis.

## Ethics Statement

This study was approved by the Ethics Committee of The Second Hospital of Jilin University (Approval No. 2024 Research Review 121) and was conducted in accordance with the Declaration of Helsinki. Because of the retrospective nature of the study and the use of deidentified patient data, the requirement for informed consent was waived by the ethics committee.

## Conflicts of Interest

The authors declare no conflicts of interest.

## Supporting information


**Supporting Information** Additional supporting information can be found online in the Supporting Information section. As an exploratory analysis, we created a nomogram based on gender + age + DBP + SBP + HbA1c + BMI + eGFR + AIP to predict the incidence of microalbuminuria in diabetic patients. The detailed materials can be found in the Supporting Information attached. There are also other detailed information not mentioned in the text, which can be found in the Supporting Information. This information can be accessed online. Supporting Information 1 Table S1: Correlation analysis of each variable with UACR and AIP. Supporting Information 2 Table S2: Baseline characteristics of participants according to AIP quartiles. Supporting Information 3 Table S3: Subgroup analysis by sex for the association between AIP and microalbuminuria. Supporting Information 4 Table S4: Subgroup analysis by obesity status for the association between AIP and microalbuminuria. Supporting Information 5 Table S5: Model fit comparisons of different lipid parameters. Supporting Information 6 Figure S1: Restricted cubic spline analysis. Supporting Information 7 Figure S2: ROC curve: AIP. Supporting Information 8 Figure S3: ROC curve_base model. Supporting Information 9 Figure S4: ROC curve_ Base Model+AIP. Supporting Information 10 Figure S5: Nomogram. Supporting Information 11 Figure S6: Calibration curve for the combined model in predicting microalbuminuria.

## Data Availability

The dataset supporting the conclusions of this article is available in the Mendeley Data repository: Liu, Yaqin (2026), “Clinical biochemical indicators data set from patients with Diabetic Nephropathy,” Mendeley Data, V1, doi:10.17632/m2mr2ffj3f.1
