# Supplementary material for: Atherogenic Index of Plasma is Independently Associated With Microalbuminuria in Type 2 Diabetes: A Retrospective Cross‐Sectional Study
Source: J Diabetes Res. 2026 Sep 8;2026:5461854. doi: 10.1155/jdr/5461854 (PMC13552219; doi:10.1155/jdr/5461854)
Supplement: Supplementary file 1 [file JDR-2026-5461854-s001.zip › 5461854.f11.docx]

**Supplementary Material 11：Calibration curve for the combined model in predicting microalbuminuria.**

The x-axis represents the predicted probability, and the y-axis represents the observed probability. The dashed diagonal line indicates perfect calibration (ideal). The blue dashed line shows the apparent performance of the model, while the solid blue line shows the bias-corrected performance estimated by 1000 bootstrap resamples. The close proximity of the bias-corrected line to the ideal line indicates good calibration. The mean absolute error (MAE) between predicted and observed probabilities was 0.031.
