# Supplementary material for: Atherogenic Index of Plasma is Independently Associated With Microalbuminuria in Type 2 Diabetes: A Retrospective Cross‐Sectional Study
Source: J Diabetes Res. 2026 Sep 8;2026:5461854. doi: 10.1155/jdr/5461854 (PMC13552219; doi:10.1155/jdr/5461854)
Supplement: Supplementary file 1 [file JDR-2026-5461854-s001.zip › 5461854.f5.docx]

The solid blue line represents the adjusted odds ratio (OR) for the outcome across the continuum of AIP values, with 4 knots placed at the 5th, 35th, 65th, and 95th percentiles of the AIP distribution. The shaded blue area indicates the 95% confidence interval. The dashed red horizontal line corresponds to OR = 1.0 (reference). The model was adjusted for sex, age, SBP, DBP, BMI, HbA1C, and eGFR. The overall association was significant (χ² = 8.63, df = 3, P = 0.0346), and the test for nonlinearity was not significant (χ² = 1.66, df = 2, P = 0.4362), indicating a linear dose-response relationship.
